# Supplementary figures and images for: Superhuman performance on sepsis MIMIC-III data by distributional reinforcement learning
Source: PLoS One. 2022 Nov 3;17(11):e0275358. doi: 10.1371/journal.pone.0275358 (PMC9632869; doi:10.1371/journal.pone.0275358)

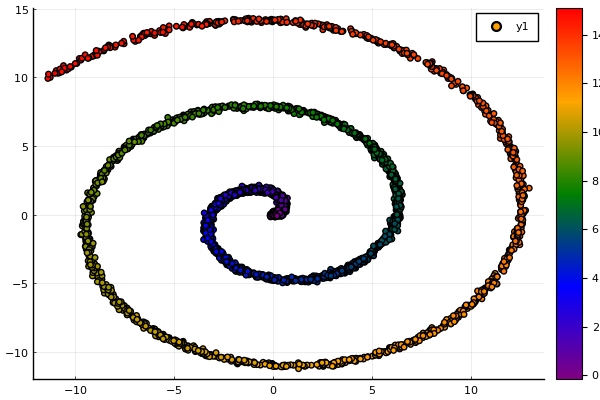

Supplement: S1 Fig — It is mapped into a 100-dimensional space using a random orthonormal mapping. (We applied the Gram-Schmidt process on a uniformly random matrix.) We then uniformly removed 70% of the observations and performed matrix completion using imputation by the mean observed value of features, kNN, and SVT [24]. (TIF) [file pone.0275358.s001.tif]

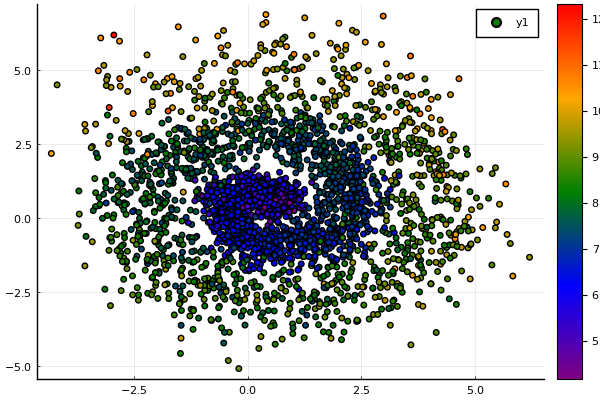

Supplement: S2 Fig — This method serves as a baseline for other completion methods. (TIF) [file pone.0275358.s002.tif]

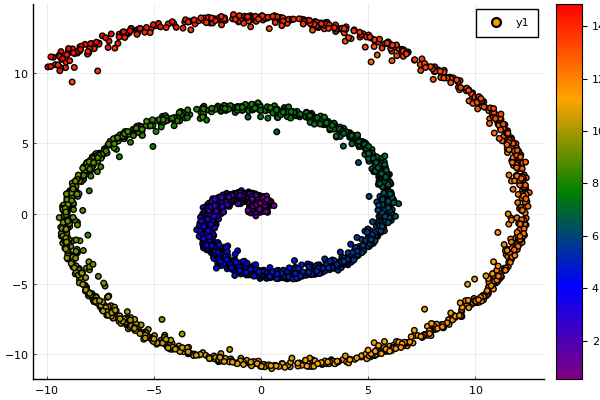

Supplement: S3 Fig — The general structure of the original data is well recovered. (TIF) [file pone.0275358.s003.tif]

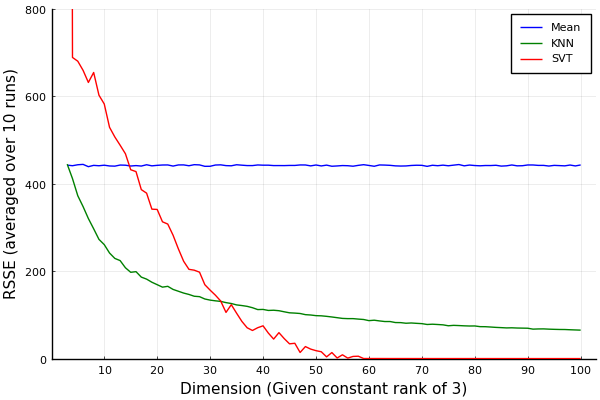

Supplement: S4 Fig — Although SVT gives an almost perfect recovery for very high dimensions, it performs very poorly (worse than mean imputation!) in low dimensions. On the other hand, the kNN recovery is always better (in terms of RSSE) than the mean imputation. (TIF) [file pone.0275358.s004.tif]
