# Supplementary material for: Superhuman performance on sepsis MIMIC-III data by distributional reinforcement learning
Source: PLoS One. 2022 Nov 3;17(11):e0275358. doi: 10.1371/journal.pone.0275358 (PMC9632869; doi:10.1371/journal.pone.0275358)
Supplement: Attachment — Submitted filename: PlosOne_Answer_Reviewers.pdf [file pone.0275358.s006.pdf]

Response Letter

Re: Manuscript ID PONE-D-21-12126
"Superhuman Performance for Sepsis Treatment by Distributional Reinforcement Learning"
Markus Böck, Julien Malle, Daniel Pasterk, Hrvoje Kukina, Ramin Hasani, Clemens Heitzinger
PLOS One

We thank the reviewers for the thoughtful and constructive feedback on our manuscript. We have worked diligently to address the suggestions and are grateful to you for your help to improve the manuscript. Our response starts with a short, high-level summary, which is followed by a point-by-point detailed answer to each question of the reviewers.

Summary. We proposed a novel and light-weighted distributional reinforcement learning (RL) algorithm for improving the Sepsis treatment. Our method uses a specialized and tractable k-nearest neighbors (kNN) for data imputation, performs clustering and decision making with a speedy distributional Q-learning. Compared to clinicians, we achieved 3% better recovery rate. We emphasize that our approach does not aim to model sepsis, but to provide a model for the clinicians' policies and to calculate an optimal treatment strategy based on the successes and errors revealed in the clinical data. Besides data preparation, our algorithm does not require any prior knowledge about the biological processes involved, since it is (implicitly) taken into account through the statistical sampling of patient data. In addition to finding optimal policies for treatments, we design a novel sepsis simulator(see Section 2.4 in our revised manuscript) that approximates the sepsis process while the patient is under treatment in the ICU.

Key Contributions:

- Ensuring the traceability and quality of the data preparation process.
- Developing an effective approach for the essential state-space approximation and using distributional RL as the learning framework.
- This framework allows us to account for risk elements in a high-stakes medical decision-support system.
- Our sepsis treatment algorithm circumvents the concerns over the applicability of the previous methods and achieves super-human performance.

Response Letter Navigation Notes:

- Reviewers' comments are highlighted in black.
- Our response to reviewers are highlighted in blue.
- Modifications in our revised manuscript have been highlighted in yellow.

Reviewer #1 general comment: The authors propose a speedy implementation of a reinforcement algorithm for sepsis. In response to Komorowski et al;. 2018, initial work, the authors propose some changes which they believe increases the performance of sepsis algorithms. The descriptions of state representation and the sepsis simulator are clear and concise. Similarly, the explanation of distributional Q-learning is very thorough and comprehensible. However there are concerns that should be addressed.
We thank Reviewer 1 for their positive evaluation of our work. We itemized the comments and provided a point-by-point response to the concerns raised.

Reviewer #1, comment #1

1. Title seems inappropriate unless the authors are reporting a randomized clinical trial demonstrating such 'superhuman' performance, I would suggest sticking with a more modest terminology.

Our response #1.1

We agree with the reviewer if we read the title from a medical perspective. The specific terminology

used in our title, however, is standard and broadly used in the context of machine learning research. Also, the title we use refers to numerical superiority within the defined framework. Additionally, conducting a randomized medical trial is extremely challenging due to the current regulations and ethical frameworks in place. That said, we do not have a reservation for this specific title and upon receiving the final decision from the reviewer and the editor we will certainly act accordingly.

Reviewer #1, comment #2

2. The decreased time-resolution to 60 minutes is nebulous and unjustified. Considering that labs, vitals, medication and other critical variables arrive at frequencies in the range of 120-250 minutes, the narrow time resolution makes me believe this algorithm is tuned to identify acute deterioration rather than sepsis. If the authors believe otherwise, please include clinical justifications.

Our response #1.2

The methodological criticism of the approach of Komorowski et al. justified the shorter time-resolution by the rapid response of the patients to sepsis treatments (particularly to vasopressors). We have now reviewed this point again and want to specify it: We cannot achieve an improvement in the detection of sepsis by increasing the temporal resolution, but we achieve an improvement in the treatment strategy with our approach. We can also state that reinforcement learning is an excellent choice in this context, because conclusions can be drawn for earlier actions even from "delayed reactions" of the environment. The increase of the temporal resolution certainly has no disadvantages in the sense of "acting".

Reviewer #1, comment #3

3. Figure 2 is difficult to follow, please characterize missingness as a function of sepsis diagnosis, it'd be interesting to see this distribution among the features that were kept out.

Our response #1.3

Missingness is almost unchanged when considering only patients that are diagnosed with sepsis, which is the reason this was not mentioned. Nevertheless, the comparison is now available in Figure 2a.

Reviewer #1, comment #4

4. Figure 2d should be recharacterized, patients that have an in-hospital death within 90 days could die of any reason, not necessarily related to sepsis. Consider in-hospital death by day 21, as it is more likely to be associated with the disease.

Our response #1.4

We agree with the reviewer but unfortunately, the feature "death by day 21" is not part of the data set.

Reviewer #1, comment #5

5. Did you evaluate whether the missingess was at random or not-at-random, i.e. did your sepsis patients have a greater prevalence of these measures than your controls, if this was the case how did you address this characterization?

Our response #1.5

Please see our Response 1.3. We have provided this information in Figure 2a.

Reviewer #1, comment #6

6. Please list the 53 variables that we retained after filtering for missingness.

Our response #1.6

We have included the table with the used features (including all 53 variables) in the supplementary materials.

Reviewer #1, comment #7

7. Figure 7a-d is missing, should probably say supplemental 1.

Our response #1.7

Thanks for denoting this. We overlooked this at the time of submission. Figure 7 indeed refers to the supplementary images. In the revised manuscript, we fixed this reference.

Reviewer #1, comment #8

8. It is a little unclear how the quantitative decision heuristic is used in determining the quality of a specific clustering profile. A figure illustrating the heuristic process in addition to the explanation would help to further elucidate the cluster quality heuristic.

Our response #1.8

This is an excellent point. We try to clarify this question in greater detail here and have updated the manuscript accordingly. As a starting point, we have the 53-dimensional vectors that constitute the episodes. We now want to create the best possible tabular problem description out of these data and reduce each of these vectors to a single state without corrupting the medical information.

In order to do this, we used the k-Means algorithm: through this algorithm, clusters are searched in such a way that the value of features that are statistically correlated in the overall data end up in the same cluster. However, there are two problems with this approach. (1) It is not clear how many clusters should be calculated (the algorithm needs the number as a parameter) in order to preserve the medical facts sufficiently well, while remaining general enough to make meaningful statements. (2) The second problem is that the algorithm is not deterministic and there is no unique solution, i.e., if the clustering algorithm is executed several times, one obtains slightly different results each time. In this regard, in the data preparation phase, we have now run the algorithm 50 times each for 19 different numbers of clusters and got a total of 950 different ways of discretizing the raw data to ensure statistical significance.

At this point, it is necessary to develop a meaningful decision criterion to select a viable solution. We define the requirements for this criterion as follows: (1) it is based exclusively on the raw data and does not anticipate any results, and (2) only the outcome of the episodes is relevant for computing the performance.

We refer to one method of implementation that we developed in this work as the "coverage heuristic." It can be motivated by the following considerations: A clustering solution is considered to be good if it does not change the vital actions provided in raw data and their resulting consequences. For instance a good heuristic for measuring this are the actions of physicians.

In the RL framework, numerical values are assigned to the respective states. In general, this is the expected value of the return. So we divide the available data into two halves and now try to determine these values for the states under the clinician policy. For this we use a method from dynamic programming and get for 600 states, for example, 600 values of how good the respective states perform under this policy. In the next step we take the second half of the data and determine the values there as well, but now not with the "own" policy, but with the clinician behavior from the first half. This is the crucial step, because if it now turns out that bad states in the first half of the data are also bad states in the second half (the same is true for good states), then this indicates that the chosen clustering is able to distinguish good and bad states under some clinician treatment.

We use this idea to compare the quality of our 950 different solutions. To do this, we look at the respective halves of the data, pairwise, determine their value under the clinician policy of the first half, and define the "coverage" as the relative frequency of numerical deviations smaller than 15. The value 15 is selected empirically based on our scale, since the values range from -100 to 100. This gives us a means of comparison that allows us to distinguish good solutions from bad ones. The selection is based exclusively on the raw data and the recorded interactions of the clinicians.

Accordingly, we have updated the manuscript and improved the discussions on clustering.

Reviewer #1, comment #9

9. Have the authors considered incorporating any short-term rewards into the simulated environment in order to improve the agent's results. For instance, perhaps there could be some reward that increases an agent's score for earlier and accurate diagnoses of sepsis.

Our response #1.9

Our algorithm with its current setup indeed goes for the shortest possible trajectory that leads to recovery in order to maximize the sum of discounted rewards (here return $\gamma^n R$). In particular, the employed discount factor of $\gamma = 0.99$ and no short-term rewards results in returns of $\gamma^n R$, where $R \in \{-100, 100\}$ and n is the length of the trajectory.

Therefore, this encourages the agent to go for the shortest possible trajectories that lead to recovery in order to maximise the return $\gamma^n R$. Identifying a concrete short-term reward structure – although challenging – is an interesting idea to explore further. Perhaps an inverse reinforcement learning algorithms can be developed for this purpose. Please also denote – as mentioned in the introduction of our manuscript – that the learning task here is not sepsis diagnosis, but to provide a model for the clinicians' policies and to calculate an optimal treatment strategy based on the successes and errors revealed in the clinical data.

Reviewer #1, comment #10

10. Since a bulk of the data used in this work is derived from time-delayed measures, the authors should discuss recent similar work in the sepsis space that utilize sensor-derived physiomarkers to see how those approaches can be used to improve the state definitions. Please see:

Alqahtani, M.F., Marsillio, L.E. and Rozenfeld, R.A., 2014. A review of biomarkers and physiomarkers in pediatric sepsis. Clinical Pediatric Emergency Medicine, 15(2), pp.177-184.

Mohammed, Akram; Van Wyk, Franco†; Chinthala, Lokesh K.*; Khojandi, Anahita†; Davis, Robert L.*; Coopersmith, Craig M.‡; Kamaleswaran, Rishikesan‡ Temporal Differential Expression of Physiomarkers Predicts Sepsis in Critically Ill Adults, SHOCK: September 28, 2020 - Volume Publish Ahead of Print - Issue - doi: 10.1097/SHK.0000000000001670*

Zimmet, A.M., Sullivan, B.A., Moorman, J.R., Lake, D.E. and Ratcliffe, S.J., 2020. Trajectories of the heart rate characteristics index, a physiomarker of sepsis in premature infants, predict Neonatal ICU mortality. JRSM cardiovascular disease, 9, p.2048004020945142.

Our response #1.10

These references are indeed very helpful to have a conclusive overview of available sources of data for better algorithm design. The overall quality of our decision-making algorithms is highly related to the quality and to the characteristics of the data. The use of biomarkers and physiomarkers suggested by the recent line of work denoted by the reviewer as an additional source of data can presumably enhance the performance of the algorithms especially in cases where a more rapid action is required. We believe that combining the time-delayed data we used here together with the suggested data collected on online testing can help develop even more performant sepsis diagnostic and treatment algorithms.

Akram et al. 2020 trained a supported vector machine classifier on data collected from active physiomarker sensor streams and successfully detected sepsis with a high rate. In our paper, as mentioned before, our aim was not to perform sepsis diagnosis, but to evaluate treatment policies using distributional RL. Our algorithm in principle is complementary to the one proposed by Akram et al. 2020. We can use the outputs of the SVM classifier as an additional input for our decision making agent to find better treatment policies.

Reviewer #1, comment #11

11. The limitations section of the manuscript is lacking, there are many limitations in this approach and it should be reported clearly.

Our response #1.11

We agree with the reviewer. Please find a summary of the limitations of our approach here which is now included in Section 5 of our manuscript: Similar to any machine learning algorithm, our algorithm is as good as the quality of the data we use for training. We would also like to mention that our approach is not intended to diagnose sepsis, but in its treatment. It turns out for us that reinforcement learning is an excellent choice in this problem context, because conclusions can be drawn for prior actions even from "delayed reactions" of the environment.

From our point of view, it is also very promising from a methodological point of view, since this approach is more holistic than the pure classification and application of predefined strategies. In essence, our approach allows also a more fine-grained sequence of actions that can be extracted from existing patient records and treatment histories.

Regarding a further limitation, of course, as with all machine learning problems, there remains the question of how much domain knowledge one may and should put into it. One point where care is needed here is clearly the reward function. In order to make the maximum possible valid statement for this problem, we intentionally decided against assigning intermediate rewards, i.e., whether patients survived or not is the only criterion. The question from which point in time this is the case, we found different information in the literature – due to lack of raw data we used the status after 90 days, which should be viable for this disease.

Reviewer #2 general comment: To improve the Sepsis Treatment for clinicians, a machine learning method, distributional reinforcement learning, is proposed in this study. The agent with the proposed method performed higher efficiency comparing to the human case. The idea and the method is very interesting, and experiment results are valid. However, I suggest authors to improve the manuscript considering the follows.

Our response: We thank Reviewer 2 for the positive evaluation of our work. At this point, we would also like to address the point raised regarding the availability of source data: The work is based entirely on free and open databases. Unfortunately, we are not allowed to share these data directly, but access can be requested at `https://physionet.org/` for reseach purposes.

We itemized the comments and provided a point-by-point response to the concerns raised.

Reviewer #2, comment #1

1. The definition of problem needs to be given more clearly. In fact, readers like a clinician or an AI researcher are confused by the novel idea. My first question is what the input space of reinforcement learning is, i.e., the concrete states, and what actions are?

Our response #2.1

Actions are represented by the volume of intravenous fluids and dose of vasopressors. The vasopressors consist of vasopressin, dopamine, epinephrine, norepinephrine and phenylephrine, while intravenous fluids consist of infusions of blood products, crystalloids, colloids and boluses. The state space is defined by using k-means clustering on time series of patient data represented with 53 features, which are all given in detail in the supplementary materials.

Reviewer #2, comment #2

2. How to evaluate the proposed method? Is it better than other reinforcement learning models? To answer this question, experiment results are needed.

Our response #2.2

We would like to stress on the fact that the advanced data processing scheme proposed here (both data preparation and state representation), is the main contributor to achieving high degrees of performance. The choice of particular RL algorithms is of less relevance here, as the differences in learning speed and data efficiency are negligible. However, the choice of the distributional reinforcement learning framework is motivated by the fact that we obtain an additional detailed insight into the decision basis for the computed policy, which is highly relevant for the type of application here.

Finally, we would like to once again thank the reviewers for their constructive suggestions. They led to significant improvements of the content and presentation of our paper.

With best regards,
Markus Böck, Julien Malle, Daniel Pasterk, Hrvoje Kukina, Ramin Hasani, Clemens Heitzinger
