# Supplementary material for: Superhuman performance on sepsis MIMIC-III data by distributional reinforcement learning
Source: PLoS One. 2022 Nov 3;17(11):e0275358. doi: 10.1371/journal.pone.0275358 (PMC9632869; doi:10.1371/journal.pone.0275358)
Supplement: Attachment — Submitted filename: Answer_Reviewers.pdf [file pone.0275358.s007.pdf]

Response Letter

Re: Manuscript ID PONE-D-21-12126
"Superhuman Performance for Sepsis Treatment by Distributional Reinforcement Learning"
Markus Böck, Julien Malle, Daniel Pasterk, Hrvoje Kukina, Ramin Hasani, Clemens Heitzinger
PLOS One

We thank the reviewers for their thoughtful and constructive feedback on our manuscript. We have addressed all suggestions and are grateful for your help in improving the manuscript. Our response starts with a short, high-level summary, which is followed by a point-by-point detailed answer to each question by the reviewers.

Summary. We proposed a novel and light-weighted distributional reinforcement learning (RL) algorithm for improving sepsis treatment. Our method uses a specialized and tractable k-nearest neighbors (kNN) algorithm for data imputation, performs clustering and decision making with a speedy distributional Q-learning. Compared to clinicians, we achieved a 3% better recovery rate. We emphasize that our approach does not aim to model sepsis, but to provide a model for the clinicians' policies and to calculate an optimal treatment strategy based on the successes and errors revealed in the clinical data. Besides data preparation, our algorithm does not require any prior knowledge about the biological processes involved, since it is (implicitly) taken into account through the statistical sampling of patient data. In addition to finding optimal policies for treatments, we have designed a novel sepsis simulator (see Section 2.4 in our revised manuscript) that approximates the sepsis process while the patient is under treatment in the ICU.

Key Contributions:

- Ensuring the traceability and quality of the data preparation process.
- Developing an effective approach for the essential state-space approximation and using distributional RL as the learning framework.
- This framework allows us to account for risk in a high-stakes medical decision-support system.
- Our sepsis treatment algorithm circumvents the concerns over the applicability of the previous methods and achieves super-human performance.

Response Letter Navigation Notes:

- Reviewers' comments are highlighted in black.
- Our response to reviewers are highlighted in blue.
- Modifications in our revised manuscript have been highlighted in yellow.

Reviewer #1 general comment: Thank you for your response. However I think you should specifically address the relevant citations in your discussions, the lack of such related works is not appropriate at this stage of the sepsis AI research.
We have addressed the references mentioned in the previous review only as an answer to the reviewers, but unfortunately we did not include them in the manuscript. Of course, this was not intended, and we have now added them. We itemized the comments and provided a point-by-point response to the concerns raised.

Reviewer #1, comment #1

1. *Can you clarify how missingness was computed, i.e., at the population level or patient level?*

Our response #1.1

Missingness was computed for each feature at the population level. This was added to Figure 2's caption for clarity.

Reviewer #1, comment #2

2. Figure 2b can be separated into sepsis and controls to see whether there are any differences.

Our response #1.2

The figure was updated with your comment: sepsis patients tend to have slightly shorter episodes (i.e. shorter stays in the ICU).

Reviewer #1, comment #3

3. Figure 2c and 2d are example patients, why were these patients selected and how is this representative of the entire sample size?

Our response #1.3

Figure 2c is not an example patient, but a 3D projection of the data corresponding to each discrete state (see Section 2.3 "State Representation"), colored according to survival rates. A reference to Section 2.3 was added to the figure's caption to avoid misunderstandings.

Figure 2d are randomly selected patients, and aims at helping the reader to visualize concrete outcomes of the sepsis simulator described in Section 2.4. We clarified that this figure is only a visual aid, for we use complete data in our computations rather than this 3D projection.

Reviewer #1, comment #4

4. I'm a bit confused on mortality/survival, why was 90 days chosen? Patients who died 90 days post meeting sepsis criteria may not have died of sepsis directly, how do you ascertain this? This needs some further investigation and justification.

Our response #1.4

We agree with this point in general, of course, but we can only use the data that are available in the large medical databases. MIMIC-III provides three pieces of information about the time of death: "death after 48h," "death in hospital," and "death after 90 days." Forty-eight hours seems to be a too short time span here. We have therefore decided that we would be more cautious and chose 90 days.

We would also like to point out that this rather conservative approach has a negative impact on the performance of the learning algorithm, since more immediate feedback from the environment has a more positive effect on the performance of the algorithms used. Under these difficult conditions, we were surprised that the method was able to produce very good performance.

During the design phase of the work, it also became clear very quickly that – as always when data are used – the quality of the database is crucial (in addition to the methods). Despite the problems with the lack of reference to direct cause of death, the choice for the MIMIC-III database still seemed the most reasonable because the quality of the measurements was still high.

Reviewer #1, comment #5

5. Again the title is inappropriate, there are no treatments (i.e drugs) for sepsis, there are only therapies (fluid resuscitation, vasoconstriction, etc.) that can reduce mortality.

Our response #1.5

We have possibly looked at the word "treatment" here with too little differentiation and overlooked the fact that it could lead to misunderstandings here from a medical point of view.

Our point of view is the following: The term "sepsis treatment" is already a term in use in the field of artificial intelligence, which has been used especially in recent years in a large number of publications. (We have here some in our references, which use this term and have been published in relevant journals.) It was therefore appropriate to follow this research and to use this term as well.

At the same time, the differentiation of this term is not of central importance to us, as the data science approach we have chosen models the "micromanagement" within a treatment as a black box and works exclusively with the abstract sequence of state – action – outcome. Using this approach, we naturally expose ourselves to some criticism, which, however, cannot be systematically eliminated, since abstraction and simplification can at best be refined, but not eliminated. Nevertheless, we believe in the large

potential of this method, since one of the essential qualities of reinforcement learning is to learn model-free and thus to evaluate actions, which may seem counter-intuitive from the point of view of a physical/chemical/biological/medical model, but have a statistically positive effect on the overall result.

To describe it with a common known comparison from RL literature: To master the game of chess, only the fall of the opponent's king is relevant. Teaching the agent which other pieces may fall along the way would negate the greatest advantage of RL.

We see ourselves in the following situation here: from a contextual point of view, the specific choice of the term "treatment" does not really matter, but we would like to connect to and reference the previous scientific work that actually uses this term. In any case, we want to pass on the decision to the editor and – if it is desired – try to find alternative suggestions.

Reviewer #1, comment #6

6. The outcome measure is also somewhat nebulous, if you define successful management as reduction in death alone, that is problematic as you in-hospital mortality statistics are wholly inappropriate for determining outcomes. I would consider you expand your outcome definition to look at concrete clinical measures, i.e. successful extubation, successful resuscitation, transfer to ward, discharge to home etc.

Our response #1.6

Our outcome measure is simply whether or not someone is alive after 90 days, which is well-defined and the most conservative one available. Of course, there may be cases where causes of death were not related to sepsis, but we assume only that someone who is still alive after 90 days must have survived the (possibly prolonged) stay in the ICU.

As mentioned in item 5 above, the major essence of our method is that we can work model-free to a large degree. Although it is technically possible to assign "intermediate rewards" with our method, this is associated with the risk of an additional softening of the definition boundaries and fundamentally undermines the scientific integrity of the method: We would then be in a medical debate about the choice of our models, and the research question would be a different one. We would then be trying to approximate medical behavior.

Reviewer #1, comment #7

7. The results section is very underwhelming, I'd expect a better reporting of the comparisons between what is deemed clinician performance (also a further explanation of how this was characterized). This again tempers my enthusiasm for the 'superhuman' performance of this algorithm if I don't quite know what humans you are comparing against.

Our response #1.7

We have revised the results section and tried to respond to the criticism as best as possible. In addition, we would like to note and summarize a few points that are relevant from our point of view:

- We are, of course, to some extent dependent on medical domain knowledge. As the essential point for us is that the mortality of sepsis patients correlates very strongly with the administration of vasopressors and intravenous fluids. Of course, this is only true if one assumes the best possible ICU treatment by the physicians. These best possible treatments can neither be replaced nor evaluated by our method. However, we have to put our scientific focus on the mentioned correlation, because we can isolate it sufficiently well in the available data and at the same time it allows us to fulfill our methodological requirements (e.g. make sure that the Markov property is preserved).

- The quality and quantity of the data collected is critical – much of the data proposed in this discourse is not available in our database and cannot be extrapolated. If the data were available, we would be happy to compare performance here. However, the essence of our approach is that we want (and need) to look at an end-to-end approach, as model-independent as possible.

- The comparison between physicians and our AI agents is difficult to define precisely: Our approach needs the certainty that the medical care in case of sepsis is performed according to state-of-the art and focused on the aspect of the mentioned drugs, because they can be extracted well from the data and there is an obvious correlation. The "superhuman performance" results from the combination

of best possible state-of-the-art medical treatment combined with optimization of a selected set of drug dosages from a large amount of statistically processable data. One argument for the choice of the MIMIC-III database was, among others, the fact that the basic data from the treatments were collected from a “teaching tertiary hospital” and we therefore assume that we can expect state-of-the art methodology here. The comparison of the survival rate in ICUs with the alternative eRI database, which also includes non-teaching hospitals, confirms this assumption (7.4% mortality MIMIC-III vs 9.8% mortality eRI).

- To address all of these methodological uncertainties and relevant criticisms best, we used a conservateve and modest definition of success. The choice of 90 days mortality degrades the operating conditions of our method significantly, which makes the result of being able to postulate a better end-to-end performance more valuable. Here we also see the greatest potential in the treatment of sepsis, since we do not want to show a revolutionary method alternative to a classical treatment approach, but would like to point out how to use obviously existing optimization potential.

Finally, we would like to once again thank the reviewers for their constructive suggestions. They led to significant improvements of the content and presentation of our paper.

With best regards,
Markus Böck, Julien Malle, Daniel Pasterk, Hrvoje Kukina, Ramin Hasani, Clemens Heitzinger
