# Supplementary material for: Superhuman performance on sepsis MIMIC-III data by distributional reinforcement learning
Source: PLoS One. 2022 Nov 3;17(11):e0275358. doi: 10.1371/journal.pone.0275358 (PMC9632869; doi:10.1371/journal.pone.0275358)
Supplement: Attachment — Submitted filename: answer_letter.pdf [file pone.0275358.s009.pdf]

Response Letter

Re: Manuscript ID PONE-D-21-12126
"Superhuman Performance on Sepsis MIMIC-III Data by Distributional Reinforcement Learning"
Markus Böck, Julien Malle, Daniel Pasterk, Hrvoje Kukina, Ramin Hasani, Clemens Heitzinger
PLOS One

We thank the reviewers for the thoughtful and constructive feedback on our manuscript. We have worked diligently to address the suggestions and are grateful to you for your help to improve the manuscript.

Summary. We proposed a novel and light-weighted distributional reinforcement learning (RL) algorithm for improving the Sepsis treatment. Our method uses a specialized and tractable k-nearest neighbors (kNN) for data imputation, performs clustering and decision making with a speedy distributional Q-learning. Compared to clinicians, we achieved 3% better recovery rate with regard to the 90-day mortality. We emphasize that our approach does not aim to model sepsis, but to provide a model for the clinicians' policies and to calculate an optimal treatment strategy based on the successes and errors revealed in the clinical data. Besides data preparation, our algorithm does not require any prior knowledge about the biological processes involved, since it is (implicitly) taken into account through the statistical sampling of patient data. In addition to finding optimal policies for treatments, we design a novel sepsis simulator(see Section 2.4 in our revised manuscript) that approximates the sepsis process while the patient is under treatment in the ICU.

Key Contributions:

- Ensuring the traceability and quality of the data preparation process.
- Developing an effective approach for the essential state-space approximation and using distributional RL as the learning framework.
- This framework allows us to account for risk elements in a high-stakes medical decision-support system.
- Our sepsis treatment algorithm circumvents the concerns over the applicability of the previous methods and achieves super-human performance.

Response Letter Navigation Notes:

- Reviewers' comments are highlighted in black.
- Our response to reviewers are highlighted in blue.
- Modifications in our revised manuscript have been highlighted in yellow.

Reviewer #1, comment #1

Thank you for your careful review and response of the revisions. Most comments have been addressed satisfactoriy. Please make mention of the your response 1.10 in the actual manuscript and emphasize this distinction in comparison to those stated works.

Our response #1.1

We have expanded the manuscript accordingly and placed the recommended sources in the appropriate context. We hope that we have been able to do this adequately well.

Finally, we would like to thank the reviewers for their constructive suggestions. They led to significant improvements of the content and presentation of our paper.

With best regards,
Markus Böck, Julien Malle, Daniel Pasterk, Hrvoje Kukina, Ramin Hasani, Clemens Heitzinger
